# Cross-cultural adaption and psychometric investigation of the German version of the Evidence Based Practice Attitude Scale (EBPAS-36D)

**DOI:** 10.1186/s12961-021-00736-8

**Published:** 2021-06-02

**Authors:** Katharina Szota, Jonathan F. B. Thielemann, Hanna Christiansen, Marte Rye, Gregory A. Aarons, Antonia Barke

**Affiliations:** 1 Department of Psychology, Philipps-University of Marburg, Gutenbergstr. 18, 35032 Marburg, Germany; 2 Department of Clinical and Biological Psychology, Catholic University of Eichstätt-Ingolstadt, Levelingstr. 7, 85049 Ingolstadt, Germany; 3 Faculty of Health Sciences, UiT The Arctic University of Norway, Regional Centre for Child and Youth Mental Health and Child Welfare, 9037 Tromsø, Norway; 4 Department of Psychiatry, University of California, 9500 Gilman Drive (0812), La Jolla, San Diego, CA 92093-0812 USA

**Keywords:** Evidence-based practice, Evidence-based treatments, Interventions, Implementation, Attitudes, Therapists, Mental health

## Abstract

**Background:**

The implementation of evidence-based practice (EBP) in mental health care confers many benefits to patients, and research into factors facilitating the implementation of EBP is needed. As an important factor affecting the implementation of EBP, service providers’ attitudes toward EBP emerged. The Evidence-Based Practice Attitude Scale (EBPAS-36) is an instrument with good psychometric characteristics that measures positive and ambivalent attitudes toward EBP. However, a German version is missing. The present study therefore aims to provide a validated German translation of the EBPAS-36.

**Methods:**

The scale was translated and back-translated as recommended by standard procedures. German psychotherapists were recruited to participate in an online survey. They provided demographic and professional information, completed the EBPAS-36, the Implementation Climate Scale (ICS) and the Intention Scale for Providers (ISP). Standard item and reliability analyses were conducted. Construct validity was evaluated with exploratory (EFA) and confirmatory factor analyses (CFA) in two subsamples (random split). Convergent validity was tested by predicting a high positive correlation of the EBPAS-36D with two scores of attitudes of the ISP and an interest in EBP score. It was tested whether the EBPAS-36D predicts the intention to use EBP.

**Results:**

*N* = 599 psychotherapists participated in the study. The item analyses showed a mean item difficulty of *p*_i _= 0.64, a mean inter-item correlation of *r* = 0.18, and a mean item-total correlation of *r*_itc_ = 0.40. The internal consistency was very good for the total scale (*α* = 0.89) and ranged from adequate to very good for the subscales (0.65–0.89), indicating high reliability. The original factor structure showed an acceptable model fit (RMSEA = 0.064 (90% CI = 0.059–0.068); SRMR = 0.0922; AIC = 1400.77), confirming the 12-factor structure of the EBPAS-36. However, a second-order factor structure derived by the EFA had an even better model fit (RMSEA = 0.057 (90% CI = 0.052–0.062); SRMR = 0.0822; AIC = 1274.56). When the EBPAS-36D was entered in a hierarchical regression model with the criterion Intention to use EBP, the EBPAS-36D contributed significantly to the prediction (Change in *R*^2^ = 0.28, *p* < 0.001) over and above gender, age and participants’ report of ever having worked in a university context.

**Conclusions:**

The present study confirms good psychometric properties and validity of a German version of the EBPAS-36 in a sample of psychotherapists.

**Supplementary Information:**

The online version contains supplementary material available at 10.1186/s12961-021-00736-8.

## Contributions to the literature

- The article provides a rigorously conducted German translation of the Evidence-Based Practice Attitudes Scale (EBPAS-36).
- The EBPAS-36D was tested in a large sample of German psychotherapists and demonstrated good item characteristics and internal consistency.
- The EBPAS-36D predicted the intention to use evidence-based practices.
- The examination of the factor structure of the EPBAS-36D advances theoretical considerations regarding the underlying constructs and cross-cultural consistency of provider’s attitudes toward EBP.

## Background

The Institute of Medicine defines evidence-based practice (EBP) as “the integration of best research evidence with clinical expertise and patient values” ([1, 2], p. 147). In order to maintain and improve the effectiveness of health care, the implementation of EBP in routine care is a major objective [3]. If successful, EBP implementation may result in several advantages, including participation in informed health care decisions [4], and better outcomes for patients [5], guidance for the development of treatment plans for practitioners [6], and increased cost-effectiveness of interventions for the health care system [7–9]. Although facilitating the uptake of EBP is in the interest of all stakeholders (including government agencies and insurance companies), a substantial gap between research and practice is evident [10]. This gap results in a large proportion of patients who receive interventions that are not justified in terms of safety, efficacy or cost-effectiveness [11–13]. Accordingly, in the past 20 years, enormous efforts have been made to disseminate and implement EBP in mental health care [14–16]. This gave rise to a rapidly growing research interest in implementation of EBP in health care [17].

Previous research efforts have identified several determinants of successful implementation of EBP. The Consolidated Framework for Implementation Research (CFIR) [18] provides a typology for the complex and interacting constructs associated with successful implementation. The major domains comprise (a) intervention characteristics, (b) outer setting, (c) inner setting, (d) characteristics of individuals and (e) the implementation process. Regarding the organizational factors of implementation success (b and c), the implementation climate of the organization in which EBP should be established is an important determinant, which can be measured with the Implementation Climate Scale [19]. Perceived barriers to adopt EBP in psychotherapy were found [20] and more positive attitudes toward EBP were linked to higher organizational support [21, 22].

The Exploration, Preparation, Implementation, Sustainment (EPIS) framework identifies provider characteristics and attitudes as important in the uptake of EBPs [23, 24]. These characteristics are most relevant in the inner context of organizations where mental health services are provided [23–25]. Specifically, attitudes can influence the initial decision to consider EBP, how it is implemented and whether it is sustained beyond the implementation phase [24, 26–28]. Demographic factors seem inconsistently related to attitudes toward EBP among mental health care providers [21, 29–33]: Higher age was associated with more positive attitudes in [34–37], with less positive ones in [38–41]; women were reported to show more positive attitudes in [34, 36–38], whereas other studies found no sex differences [26, 39, 40].

An important part of the implementation research agenda is the development of pragmatic measures capturing potentially important implementation determinants, mechanisms, and outcomes [42, 43] that promote or obstruct dissemination and implementation [44]. Martinez, Lewis and Weiner [45] identify several challenges for such instruments including the use of frameworks and theoretical models including consistent construct definitions and appropriate assessments of psychometric properties [45].

The Evidence-Based Practice Attitude Scale (EBPAS) [26] is an instrument that has been identified as a psychometrically strong measure assessing positive as well as ambivalent attitudes toward EBP [26, 34, 44]. It was developed specifically for the target group of mental health care providers, but has since been employed in broader contexts [42]. In line with suggestions put forward in the literature [44, 46], it is based on mental health dissemination and implementation theories [47–49] and has been developed in collaboration with service providers and researchers [26, 27, 50]. The original 15-item version showed strong psychometric properties including high validity in various settings and samples from the US, Norway, Greece and the Netherlands [34, 37, 38, 40]. In an effort to incorporate additional relevant dimensions, the 15-item EBPAS was expanded to 50 items and 12 dimensions through formative work, which included researcher input, focus groups with program managers and clinicians, and subsequent data reduction [27]. In the interest of rendering the measure more brief and pragmatic [46], the 50-item version was reduced to 36-items while retaining 12 dimensions [52]. On the one hand, the domains assess positive attitudes toward EBP: the intuitive Appeal of EBP, the willingness to adopt EBP given the Requirements to do so, providers’ Openness to new practices and manualized interventions, the Fit of EBP with values and needs of providers and clients, and providers’ perceptions of an increased Job Security provided by learning EBP, of the Organizational Support for learning EBP and of receiving Feedback [27]. On the other hand, the following domains assess ambivalent attitudes toward EBP: the Divergence between research-based interventions and current practice, the Limitations of EBP due to not addressing client needs, negative perceptions of Monitoring by supervisors, the perceived Balance of clinical skills and science in therapy, and the Burden of learning EBP [27]. The EPBAS-36 has shown good psychometric properties and cross-cultural validity in US and Norwegian samples [51].

As argued by Kien et al. [52], German instruments assessing implementation science constructs are scarce and psychometric properties rarely reported. This stands in contrast with the growing relevance and increasing efforts of implementation research in German-speaking countries [53–58]. Over the last decades, psychotherapy in these countries has experienced a significant professionalization and focus on EBP. These countries may benefit from reliable and valid instruments in implementation science. In Germany, a law (‘Psychotherapeutengesetz’) regulates the practice of psychotherapy since 1999, stating that only state approved practitioners may offer treatment [59–61]. In September 2019, the German parliament approved an adapted law that aims to further align the postgraduate training for psychotherapy to the structure of medical education [59]. Learning about German psychotherapists’ attitudes toward EBP may help to inform the psychotherapy training. To the best of our knowledge, two independent German translations of EBPAS-15 exist [62, 63], but no translation of the EBPAS-36 is available. Therefore, the present study aims to present a German translation of the EBPAS-36 and evaluate its psychometric properties.

## Methods

### Ethics

The cross-sectional online survey study was approved by the Internal Review Board of the University of Marburg (approval number: 2019-58 k). Participants received study information and provided informed consent before they were able to access the survey. Data were collected anonymously. All raw data were stored securely at the Department of Clinical Child and Adolescent Psychology at Philipps University in Marburg, Germany.

### Participants

Eligible participants were licensed psychotherapists and psychiatrists for adults, children and adolescents as well as psychotherapists and psychiatrists enrolled in postgraduate training to obtain such a license. No exclusion criteria were applied.

### Procedure

*Translation.* The translation was carried out in accordance with the WHO recommendations (www.who.int/substance_abuse/research_tools/translation/en/), including the following steps: (1) Forward translation, (2) Expert panel back-translation, (3) Pre-testing and cognitive interviewing, and (4) Final version. The EBPAS-36 [51] was translated into German by the second author (JT) (step 1) and back-translated by the bilingual English-speaking senior author (AB). The back-translation was reviewed by the original authors of the scale (MR, GAA) who provided feedback to assure the items represented the meaning and original constructs (step 2). The original and back-translated versions were then reviewed in a consensus meeting of the translating authors. Relevant items of previously available German translations of the EBPAS-15 were compared and the translations showed a good match. The consensus version was then reviewed by a group of German clinical psychotherapists (in training) and researchers (*n* = 26) as well as a graduate linguist for comprehensibility and wording (step 3). Their revisions were discussed and considered by the translating authors in a second consensus meeting, resulting in a final German version of the scale (step 4) (see Additional file 1, Additional file 2).

*Recruitment and data collection.* Data were collected (14/11/2019–27/04/2020) via an openly accessible online survey, using the scientific survey platform SoSci Survey (www.soscisurvey.de). The link was widely distributed via e-mail lists of professional psychotherapy organizations that all licensed psychotherapists are members of, universities, training institutes, and psychiatric in- and outpatient institutions as well as Facebook groups of psychotherapists and psychiatrists. On the first page of the survey, potential participants received study information and were required to provide informed consent before they were able to proceed with the survey. Additional information on the survey is found in the 'Checklist for Reporting Results of Internet E-Surveys' (CHERRIES) [64] in the Additional file 3.

### Measures

*Demographics and information on training and profession.* Participants gave standard demographic information and professional information (university degree, license status, therapy orientation, and current occupation).

*Evidence Based Practice Attitudes Scale (EBPAS-36D).* The EBPAS-36D is an instrument to assess mental health providers’ attitudes toward adopting EBP [51]. The 36 items of the EBPAS-36 load on 12 subscales of three items each: Requirements, Appeal, Openness, Divergence, Limitations, Fit, Monitoring, Balance, Burden, Job security, Organizational support, and Feedback. Respondents are asked to rate their agreement with statements on a 5-point Likert scale ranging from 0 (‘not at all’) to 4 (‘to a very great extent’). Most items are worded in such a way that a higher total score indicates a more positive attitude toward the adoption of EBP; 15 items are scored reversely. A mean of the subscales can be computed to create a total scale. The German instrument can be found in the Additional file 1, Additional file 2.

*Implementation Climate Scale (ICS).* The ICS is an 18-item instrument measuring the implementation climate in organizations and work groups [19]. The original English version was translated into German by the first author (KS) and back-translated by the bilingual English-speaking senior author (AB). In order to adapt the scale for psychotherapists in private practice, a parallel version was constructed that captures the implementation climate in the health system. Respondents are asked to rate their agreement with statements describing how the respondents perceive the climate in the institution they work at with regard to the implementation of evidence-based interventions. A 5-point Likert scale ranging from 0 (‘not at all’) to 4 (‘to a very great extent’) was used. Six subscales can be calculated: Focus on EBP, Educational Support for EBP, Recognition for EBP, Rewards for EBP, Selection for EBP, and Selection for Openness toward EBP. Means of the subscales are computed to create a total scale. In the present study, the internal consistency for the ICS regarding organizations was Cronbach’s *α* = 0.91 for the total scale and between *α* = 0.77 (Selection for Openness) and *α* = 0.92 (Focus on EBP) for the subscales. For the ICS with respect to the health system, Cronbach’s *α* was *α* = 0.90 for the total scale and between *α* = 0.77 (Rewards for EBP) and *α* = 0.88 (Educational Support for EBP) for the subscales. (A separate manuscript for this measure is in preparation.)

*Intention Scale for Providers (ISP).* The ISP is a 70-item instrument assessing individual behavioral intentions for EBP use [65] based on the theory of planned behavior [66]. The original English version was translated into German by the first author (KS) and back-translated by the bilingual English-speaking senior author (AB). Responses are given on 7-point and 4-point rating scales. Seven subscales can be calculated. Direct measurement scales of attitudes (A-D, 5 items, *α* = 0.69), subjective norms (SN-D, 3 items, *α* = 0.85), perceived behavioral control (PBC-D, 4 items, *α* = 0.69) and behavioral intention (BI-D, 4 items, *α* = 0.89) are generated by calculating the average subscale scores. Indirect measurement scales of attitudes (A-ID, 22 items, *α* = 0.80), subjective norms (SN-ID, 18 items, *α* = 0.89) and perceived behavioral control (PBC-ID, 14 items, *α* = 0.89) are created by multiplying and summing up the belief and influence items (e.g., Normative Beliefs and Motivation to Comply).

*Global assessments*. As a subjective self-assessment measure, participants were asked to rate their interest in EBP on visual analogue scales for nine questions, e.g. “How great is your interest in evidence-based treatment methods?” (see all items in Additional file 4). A total score was computed (*α* = 0.84). At the end of the survey, participants were asked to rate the honesty of their responses (‘How honestly did you answer the questions of this study?’) and their self-reported tendency toward social desirability when answering the survey (‘Did social desirability play a role in the survey?’) on visual analogue scales.

Before answering the EBPAS-36D, ICS and ISP, participants were provided the following definition of evidence-based methods: “Evidence-based methods are treatment or intervention methods (in psychotherapy, e.g., certain therapy manuals; in physical medicine, e.g., medications or surgical procedures) whose effectiveness has been empirically demonstrated in various scientific studies. This can be done, for example, by demonstrating the efficacy of a psychotherapy over that of a waiting list condition or an alternative treatment.”

### Statistical analysis

All statistical analyses were performed using IBM SPSS 26 for Windows (Chicago, IL, USA). For the confirmatory factor analysis (CFA), SPSS AMOS version 26.0.0 was used. *P* values < 0.05 were set as thresholds for statistical significance in all analyses. For the EBPAS-36D, means were computed if there was a maximum of one missing item per scale. Otherwise, respondents were excluded from analyses. For item analyses, item difficulties, corrected item-whole correlations and Cronbach’s alpha if item is deleted were calculated. To obtain internal reliability coefficients of the scales and subscales, Cronbach’s alpha was calculated. Values above 0.70 are regarded as acceptable, higher than 0.80 as good, higher than 0.90 as excellent. In order to assess construct validity, the factorial structure of EBPAS-36D was investigated by dividing the total sample randomly into two samples: With the first subsample, we conducted an exploratory factor analysis (EFA), followed by a confirmatory one (CFA) with the other subsample. Differences between both samples regarding age, gender distribution and the EBPAS-36D total scale and subscales were examined with independent *t*-tests. The suitability of data for EFA was assessed with the Kaiser-Meyer-Oklin (KMO) sample adequacy measure [67, 68] and Bartlett’s test [69]. To determine the number of components for the EFA, Horn's parallel analysis and Velicer's MAP test were conducted using the SPSS programs available online (https://people.ok.ubc.ca/brioconn/nfactors/nfactors.html) and the results compared [70]. Since parallel analysis of principal factor analysis tends to over-extract factors [71], parallel analysis of principal component analysis was conducted with raw data permutation and 1000 datasets. The EFA was conducted using principal axis factoring analysis with promax correlated factors rotation method. Subsequently, a CFA was conducted to test and compare the original 12-factor structure of EBPAS-36 against a second-order factor structure derived by the EFA, merging the EFA components 4 (Constraints by the institution), 5 (Monitoring) and 6 (Burden) into one second-order factor, and another second-order factor solution that was proposed by Rye et al. [41]. Maximum likelihood estimations were used. Since the Mardia-test for multivariate normal distribution is significant (*z* = 19.16) and all variables exceed either the limits for skewness or for excess as postulated by West et al. ([72], skewness < 2, excess < 7), an increased *χ*^2^ value was expected and the Bollen-Stine bootstrap procedure (1000 samples) was performed. The chi-square test statistic the *χ*^2^/*df* ratio, the root mean square error of approximation (RMSEA), the standardized root mean squared residual (SRMR), the comparative fit index (CFI) and the parsimony-adjusted comparative fit index (PCFI) were reported as fit indices. To assess the convergent validity of EBPAS-36D, the following hypotheses were tested by calculating Pearson correlation coefficients: The EBPAS-36D total scale shows a high positive correlation with the ISP direct and indirect scale scores of attitudes (A-D, A-ID), as well as the interest in EBP score. According to Cohen [73], *r* = 0.50 indicates high correlations. To test whether the EBPAS-36D total scale is an incremental predictor of the direct scale of behavioral intention to use EBP of the ISP (BI-D), a hierarchical linear regression analysis with the method ENTER was conducted. Gender and age (block 1), having ever worked in science (block 2), and the EBPAS-36D total scale (block 3) were successively included in the regression model to assess incremental improvements of model fit. Mean differences across gender and professional groups on the EBPAS-36D were assessed with independent *t*-tests. Pearson coefficients were calculated to assess correlations between age as well as demographic/professional variables and the EBPAS-36D. Lastly, Pearson correlations between ICS and EBPAS-36D were assessed. The findings are reported following the STrengthening the Reporting of OBservational studies in Epidemiology (STROBE) guideline [74] and informed by the COnsensus-based Standards for the selection of health Measurement INstruments (COSMIN) taxonomy [75].

## Results

### Participants

The link to the online survey was clicked 2.417 times. Overall, 913 participants continued after informed consent. Of these, 863 met the inclusion criteria (i.e., profession). A total of 261 participants were excluded due to drop-out before completion of the EBPAS-36D, two due to implausible answers (for example being 99 years old), one due to conspicuous response patterns in EBPAS-36D (e.g., straight-lining despite reverse coded items). Of the remaining 599 participants, 502 were female (83.8%) and their age ranged from 23 to 82 years (*M* = 36.62, *SD* = 11.26). Roughly half of the sample (56.26%) stated being in postgraduate training to become psychotherapists or psychiatrists. 42.7% of the German psychotherapists reported ever having worked in science. Further information on profession is presented in Table 1.

**Table 1:** Demographics and information on profession

Therapy orientation	%	Professional group	%	Current occupation	%
Cognitive Behavior therapy (CBT)	74.5	Psychotherapist in training	40.7	Outpatient practice	64.1
Psychodynamic psychotherapy (PDT)	14.9	Licensed psychotherapist	26.9	Psychiatric hospital	15.1
PDT and psychoanalytic therapy	4.3	Child and adolescent psychotherapist	16.2	Clinic for psychosomatic medicine	6.5
CBT and systemic therapy	2.2	Child and adolescent psychotherapist in training	15.4	Rehabilitation clinic/center	5.0
Other	4.1	Other	0.8	Psychiatric day-clinic	4.5
				University	2.2
				Other	2.6

### Item analysis

Detailed information on valid *n* and missing values for the EBPAS-36D items are found in Table 2. Item difficulties of EBPAS-36D ranged between *p*_i_ = 0.21 (item 28) and *p*_i_ = 0.93 (item 13) with a mean difficulty of *p*_i_ = 0.64. The mean inter-item correlation was *r* = 0.18. The item-total correlations of the individual items with the total scale ranged from *r*_itc_ = 0.07 (item 26) to *r*_itc_ = 0.62 (item 2) with a mean item-total correlation of *r*_itc_ = 0.40. Eight items showed item-total correlations under 0.30 (see Table 2). Considering the subscales, the correlations of the individual items with their subscales ranged from *r*_itc_ = 0.44 (item 5) to *r*_itc_ = 0.87 (item 9 and 29).

**Table 2:** Item analyses of EBPAS-36D

Item	Short description	*M* (*SD*)	*p*_i_	*r*_itc total_	*α*_total if deleted_	*r*_itc subscale_	*α*_subscale if deleted_	Valid *n*	Missing values (%)
1	Like to use new therapy/interventions	2.85 (0.80)	0.71	0.358	0.89	0.516	0.73	599	0 (0.00)
2	Will follow a treatment manual	2.75 (1.05)	0.69	0.617	0.88	0.621	0.62	599	0 (0.00)
3	Will try therapy/interventions developed by researchers	2.99 (0.82)	0.75	0.572	0.88	0.615	0.62	599	0 (0.00)
4 (r)	Research based treatments/interventions not useful	3.22 (0.93)	0.81	0.404	0.89	0.473	0.55	599	0 (0.00)
5 (r)	Clinical experience more important	1.65 (1.06)	0.41	0.479	0.88	0.441	0.59	599	0 (0.00)
6 (r)	Would not use manualized therapy/interventions	3.29 (1.03)	0.82	0.551	0.88	0.479	0.53	599	0 (0.00)
7	Makes sense	3.58 (0.63)	0.90	0.374	0.89	0.449	0.68	599	0 (0.00)
8	Supervisor required	2.14 (1.10)	0.54	0.520	0.88	0.841	0.79	599	4 (0.67)
9	Agency required	2.17 (1.09)	0.54	0.501	0.88	0.870	0.76	599	3 (0.50)
10	State required	2.26 (0.96)	0.56	0.421	0.89	0.655	0.94	599	1 (0.17)
11	Colleagues happy with therapy	2.92 (0.89)	0.73	0.429	0.89	0.517	0.59	599	2 (0.33)
12	Enough training	3.26 (0.86)	0.82	0.532	0.88	0.583	0.49	599	1 (0.17)
13	Right for your clients	3.71 (0.62)	0.93	0.329	0.89	0.506	0.58	599	1 (0.17)
14	Had a say in how to use the evidence-based practice	3.22 (0.90)	0.81	0.288	0.89	0.478	0.63	599	1 (0.17)
15	Fit with your clinical approach	3.51 (0.74)	0.88	0.272	0.89	0.522	0.54	599	1 (0.17)
16 (r)	Clients with multiple problems	2.48 (1.07)	0.62	0.410	0.89	0.588	0.83	581	2 (0.34)
17 (r)	Not individualized	2.35 (1.11)	0.59	0.474	0.88	0.714	0.71	581	2 (0.34)
18 (r)	Too narrowly focused	1.99 (1.10)	0.50	0.514	0.88	0.718	0.70	581	2 (0.34)
19 (r)	Work without oversight	2.06 (1.22)	0.52	0.363	0.89	0.688	0.60	581	2 (0.34)
20 (r)	Looking over my shoulder	2.18 (1.23)	0.55	0.292	0.89	0.626	0.67	581	2 (0.34)
21 (r)	My work does not need to be monitored	1.90 (1.22)	0.48	0.318	0.89	0.513	0.27	581	2 (0.34)
22 (r)	Positive outcome is an art	2.73 (0.99)	0.68	0.489	0.88	0.524	0.46	581	2 (0.34)
23 (r)	Therapy is an art and a science	1.24 (1.14)	0.31	0.175	0.89	0.432	0.59	581	2 (0.34)
24 (r)	Overall competence is more important	1.38 (1.02)	0.35	0.437	0.89	0.422	0.59	581	2 (0.34)
25 (r)	Don’t have time to learn anything new	2.90 (1.06)	0.73	0.133	0.89	0.628	0.76	581	2 (0.34)
26 (r)	Can’t meet other obligations	3.02 (1.01)	0.76	.067	0.89	0.726	0.67	581	5 (0.86)
27 (r)	How to fit evidence-based practice in	2.87 (1.14)	0.72	0.199	0.89	0.618	0.78	581	2 (0.34)
28	Help me keep my job	0.83 (1.11)	0.21	0.431	0.89	0.653	0.95	581	5 (0.86)
29	Help me get a new job	1.52 (1.32)	0.38	0.538	0.88	0.870	0.76	581	3 (0.52)
30	Make it easier to find work	1.50 (1.32)	0.38	0.522	0.88	0.850	0.78	581	4 (0.69)
31	Continuing education credits provided	2.25 (1.33)	0.56	0.470	0.88	0.642	0.88	581	4 (0.69)
32	Training provided	2.54 (1.20)	0.64	0.587	0.88	0.806	0.71	581	3 (0.52)
33	Ongoing support provided	2.41 (1.17)	0.60	0.549	0.88	0.728	0.79	581	3 (0.52)
34	Enjoy feedback on performance	2.92 (0.87)	0.73	0.312	0.89	0.574	0.71	581	4 (0.69)
35	Feedback helps me to be better	3.28 (0.82)	0.82	0.380	0.89	0.709	0.53	581	3 (0.52)
36	Supervision helps me to be better	3.59 (0.69)	0.90	0.214	0.89	0.511	0.76	581	3 (0.52)

*p*_i_: item difficulty, *r*_itc_ corrected item-whole correlation. (r): Item to be reverse scored

### Reliability

The internal consistency of the EBPAS-36D total scale was *α* = 0.89 and would not have benefitted from removing any item. Internal consistencies of the EBPAS-36D subscales were Requirements *α* = 0.89; Appeal *α* = 0.69; Openness *α* = 0.75; Divergence *α* = 0.65; Limitations *α* = 0.82; Fit *α* = 0.68; Monitoring *α* = 0.77; Balance *α* = 0.65; Burden *α* = 0.81; Job security *α* = 0.89; Organizational support *α* = 0.85; Feedback *α* = 0.76.

### Subscale correlations

The correlation coefficients between the EBPAS-36D total scale and the 12 subscales are presented in Table 3. The highest correlation was between the total scale and the Openness subscale (*r* = 0.689). On subscale level, high correlations were between the Appeal and Fit subscales (*r* = 0.609), the Divergence and Limitations subscales (*r* = 0.550), the Openness and Divergence subscales (*r* = -0.531), the Appeal and Openness subscales (*r* = 0.514) and the Job Security and Organization Support subscales (*r* = 0.547).

**Table 3:** Pearson correlations of EBPAS-36D total scale, EBPAS-36D subscales and age

	2	3	4	5	6	7	8	9	10	11	12	13	Age
1 EBPAS-36D Total scale	0.586**	0.618**	0.689**	− 0.677**	− 0.602**	0.427**	− 0.471**	− 0.536**	− 0.232**	0.613**	0.665**	0.425**	− 0.441**
2 EBPAS-36D Requirements	–	0.465**	0.359**	− 0.281**	− 0.177**	0.261**	− 0.135**	− 0.199**	0.016	0.411**	0.376**	0.151**	− 0.410**
3 EBPAS-36D Appeal		–	0.514**	− 0.376**	− 0.208**	0.609**	− 0.091*	− 0.127**	− 0.002	0.289**	0.436**	0.286**	− 0.376**
4 EBPAS-36D Openness			–	− 0.531**	− 0.359**	0.340**	− 0.113**	− 0.281**	− 0.082*	0.391**	0.468**	0.261**	− 0.424**
5 EBPAS-36D Divergence				–	0.550**	− 0.214**	0.203**	0.452**	0.119**	− 0.258**	− 0.393**	− 0.096*	0.230**
6 EBPAS-36D Limitations					–	− 0.081	0.256**	0.445**	0.185**	− 0.251**	− 0.267**	− 0.059	0.160**
7 EBPAS-36D Fit						–	− 0.034	− 0.032	− 0.088*	0.107**	0.252**	0.272**	− 0.176**
8 EBPAS-36D Monitoring							–	0.350**	0.182**	− 0.151**	− 0.120**	− 0.264**	0.137**
9 EBPAS-36D Balance								–	0.126**	− 0.179**	− 0.220**	− 0.018	0.182**
10 EBPAS-36D Burden									–	0.113**	0.168**	− 0.063	− 0.172**
11 EBPAS-36D Job security										–	0.547**	0.223**	− 0.371**
12 EBPAS-36D Org. support											–	0.291**	− 0.388**
13 EBPAS-36D Feedback												–	− 0.232**

*n* = 574–599. EBPAS-36D: German version of the Evidence-Based Practice Attitudes Scale *p < 0.05 **p < 0.001

### Validity

For analyses regarding factorial validity, 25 (4.2%) of the participants were excluded as they had more than one missing item in at least one subscale of the EBPAS-36D, so that no means could be calculated. Of those 25 excluded participants, 80.0% dropped out of the survey during the EBPAS-36D after item 15. Of those included in analyses (*n* = 574), six participants (1.0%) had missing information on only one item of EBPAS-36D, none had more than two items missing. More than 98.9% of the included participants answered all items. Split samples for EFA and CFA showed no group differences on mean age, gender, and EBPAS-36D total scale or subscales (see Additional file 5).

*EFA.* The sample adequacy measure (KMO = 0.844) and significant Bartlett’s test (*χ*^2^ (630) = 5616.83, *p* < 0.001) indicated suitability of data (*n* = 296) for analysis. Parallel analysis and MAP test both recommended the extraction of six factors. The six extracted factors accounted for 57.56% of variance. The rotated factor matrix is found in the Additional file 6. Eleven items loaded on factor 1 and explained 24.33% of the variance (factor loadings from 0.357 to 0.844). Ten items loaded on factor 2 and explained 9.41% of the variance (0.410 to 0.773). Factor 3 comprised six items that explained 7.92% of the variance (0.343 to 0.969). Three items loaded on factor 4 and explained 5.93% of the variance (0.735 to 0.946). Three items loaded on factor 5 and explained 5.27% of the variance (0.549 to 0.815). Factor 6 comprises three items that explained 4.71% of variance (0.693 to 0.841). With the exception of item 2 of the Openness subscale, items of the original subscales loaded on the extracted factors together. Item 3 of the Openness subscale showed nearly equally high factor loadings on factor 1 (0.357) and factor 2 (-0.335).

**Fig. 1:**
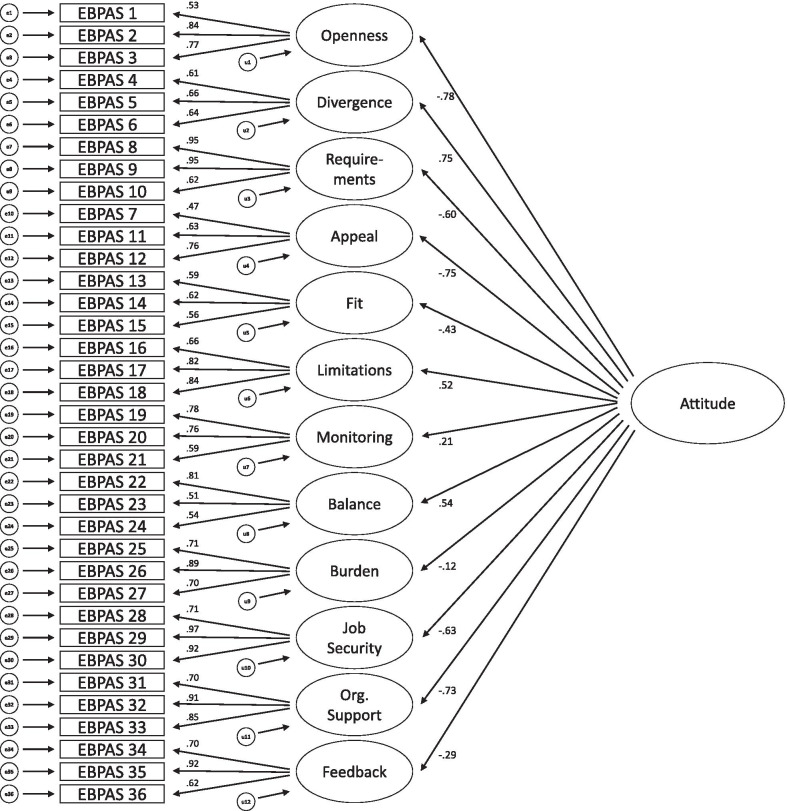
Model A: original 12-factor structure

**Fig. 2:**
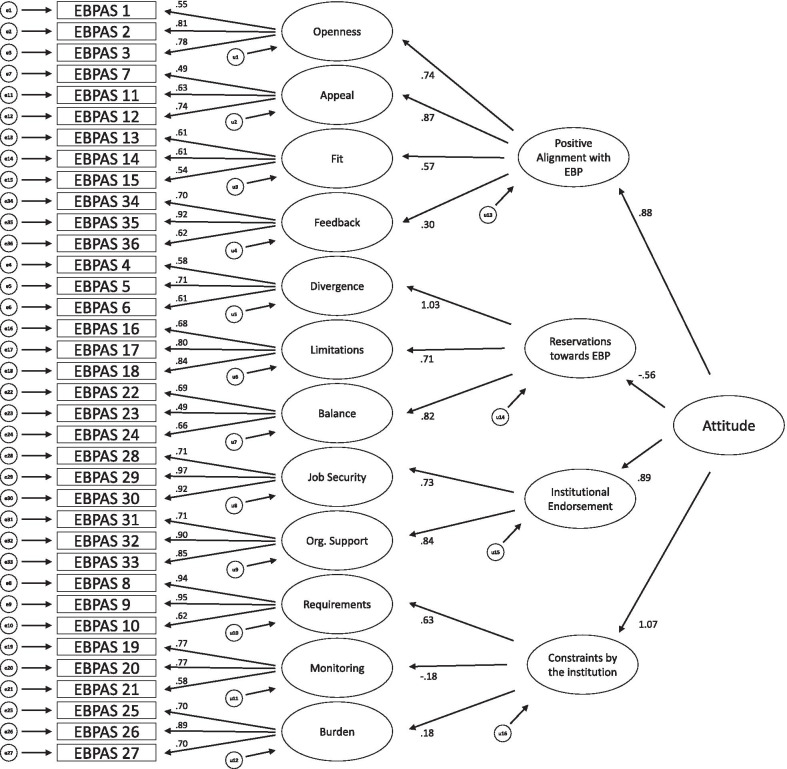
Model B: second-order 4-factor structure derived by EFA

**Fig. 3:**
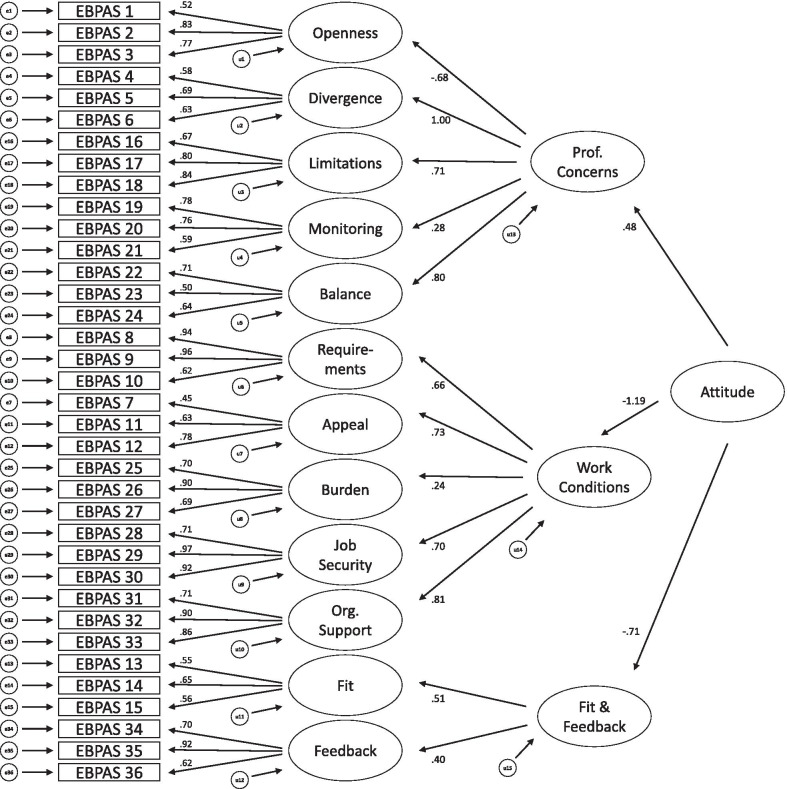
Model C: second-order 3-factor model established by Rye et al. [41]

*CFA.* The path diagrams of the original 12-factor structure (model A), a second-order 4-factor structure derived by the EFA (model B) and the second-order 3-factor model established by Rye et al. [41] (model C) are shown in Figs. 1, 2 and 3, respectively. Although the model fit of the original factor structure was adequate, both second-order models showed even better model fits (see Table 4). For all three models, all regression weights were significant.

**Table 4:** CFA: model fit indices

Model	χ^2^	*df*	*p*	χ^2^/*df*	RMSEA [90% CI]	SRMR	CFI	PCFI	AIC
A	1232.77	582	0.001^a^	2.12	0.064 [0.059; 0.068]	0.0922	0.856	0.791	1400.77
B	1098.56	578	0.001^a^	1.90	0.057 [0.052; 0.062]	0.0822	0.885	0.812	1274.56
C	1121.06	579	0.001^a^	1.94	0.058 [0.053; 0.063]	0.0857	0.880	0.809	1295.06

*n* = 278. Model A: Original 12-factor model. Model B: Second-order model derived by EFA. Model C: Second-order model by Rye et al. a: Bollen-Stine-corrected. *RMSEA* root mean square error of approximation, *SRMR* standardized root mean residual, *CFI* comparative fit index, *PCFI* parsimony-adjusted CFI, *AIC* Akaike information criterion

*Correlation analyses.* The EBPAS-36D total scale correlated with the direct scale of attitudes (A-D, *r* = 0.663, *p* < 0.001, *n* = 574) and the indirect scale of attitudes (A-ID, *r* = 0.531, *p* < 0.001, *n* = 126) of the ISP. The global 9-item assessment of participant’s interest in EBP correlated with the EBPAS-36D total scale (*r* = 0.529, *p* < 0.001, *n* = 566).

*Regression analysis.* The EBPAS-36D total scale was included in a hierarchical regression model to predict the Behavioral Intention Scale of the ISP as a third block, subsequent to the predictors gender and age (block 1) and ever having worked in science (block 2). The inclusion improved the model fit (Change in *R*^2^ = 0.28, *F* = 267.32, *p* < 0.001) and the significant regression coefficient of the EBPAS-36D total scale (*ß* = 2.13; *t* = 16.35; *p* < 0.001) indicated incremental prediction beyond the previous predictors (see Tables 5 and 6).

**Table 5:** Model summary

Model	*R*^2^	*R*^2^_corr_	*SE*	*F*	*df*	*p*	Change in *R*^2^	*F*	*p*
1	0.098	0.095	1.54	29.30	2; 540	< 0. 001			
2	0.156	0.151	1.49	33.17	3; 539	< 0.001	0.058	37.01	< 0.001
3	0.436	0.432	1.22	103.99	4; 538	< 0.001	0.280	267.32	< 0.001

*n* = 543. Dependent variable: Behavioral Intention Scale of Intention Scale for Providers. Model 1: Gender, Age. Model 2: Gender, Age, Work in Science. Model 3: Gender, Age, Work in Science, EBPAS-36D total scale

**Table 6:** Regression coefficients

Model		ß	95% CI of ß	*SE*	*t*	*p*
1	(Constant)	7.55	6.98; 8.13	0.29	25.78	< 0.001
	Gender	0.12	− 0.24; 0.47	0.18	0.65	0.519
	Age	− 0.05	− 0.06; − 0.04	0.01	− 7.64	< 0.001
2	(Constant)	8.81	8.12; 9.50	0.35	25.11	< 0.001
	Gender	0.03	− 0.31; 0.37	0.17	0.16	0.873
	Age	− 0.05	− 0.06; − 0.03	0.01	− 7.47	< 0.001
	Work in Science	− 0.79	− 1.05; − 0.54	0.13	− 6.08	< 0.001
3	(Constant)	1.22	0.14; 2.29	0.55	2.23	0.026
	Gender	0.17	− 0.11; 0.45	0.14	1.18	0.237
	Age	− 0.01	− 0.02; 0.00	0.01	− 2.02	0.044
	Work in Science	− 0.35	− 0.56; − 0.13	0.11	− 3.27	0.002
	EBPAS-36D total scale	2.13	1.88; 2.39	0.13	16.35	< 0.001

*n* = 543. Dependent variable: Behavioral Intention Scale of Intention Scale for Providers

### Group differences and correlations

Compared to women, men scored lower on the EBPAS-36D total scale (*t* (570) = 2.59; *p* = 0.010) and the subscales Requirements (*t* (592) = 2.91; *p* = 0.004), Appeal (*t* (595) = 3.73; *p* < 0.001), Fit (*t* (595) = 2.91; *p* = 0.004) and Organizational Support (*t* (579) = 2.66; *p* = 0.008). Age was correlated with the EBPAS-36D total scale and all subscales (see Table 3), indicating more negative attitudes toward EBP with increasing age. Consistent with this, licensed psychotherapists differed from psychotherapists in training on all subscales and the total scale of the EBPAS-36D (*t* (445.82) = − 7.581; *p* < 0.001; *d* = − 0.72), indicating more positive attitudes of psychotherapists in training, which is consistent with prior findings regarding professional development level and attitudes toward EBP [26].

The EBPAS-36D total scale was associated with the total score of the ICS on organizations and work groups (*r* = 0.432, *p* < 0.001, *n* = 408), but not with the total scale of the ICS on the health system (*r* = 0.138, *p* = 0.080) that was exclusively completed by licensed psychotherapists and psychiatrists working in private practices (*n* = 161). The self-rated honesty when answering the survey showed a small correlation with the EBPAS-36D total scale (*r* = 0.146, *p* = 0.001, *n* = 556).

## Discussion

The present study is the first to present a German version of the EBPAS-36 and investigate its validity and psychometric properties. In a sample of psychotherapists and psychiatrists, the original factor structure was confirmed and the EBPAS-36D demonstrated good item properties, internal consistency and convergent validity.

Rising implementation research efforts in German-speaking countries necessitate the development and psychometric examination of German instruments assessing implementation research constructs [52]. Regarding characteristics of individuals, additional well-suited instruments have been translated and validated during the course of our study, for example the Evidence-based Practice Inventory (EBPI) questionnaire [76]. The EBPI assesses health care providers’ adherence to EBP as well as barriers and facilitators for the use of EBP. A total of 26 items load on five domains: attitude, subjective norm, perceived behavioral control, decision making and intention and behavior. A German version was adopted and its reliability was examined in a nationwide online survey [76]. The EBPI and EBPAS-36D could thus complement each other well in future implementation research studies, with the latter focusing on attitudes and capturing diverse aspects of positive and ambivalent attitudes toward EBP. The evaluation of providers’ attitudes toward EBP with help of both instruments might inform about successful strategies in implementation efforts in research in German-speaking countries as well as potential targets for improvement in clinical training and practice. For example, The Leadership and Organizational Change for Implementation (LOCI) strategy can be used to improve workplace climate for EBP, which should then influence provider attitudes toward, and use of, EBP with fidelity [77, 78]. Thus, attitudes could be considered a mechanism by which an implementation strategy has its effects on clinical practice. This is consistent with an implementation science approach where it is recommended to identify and integrate the use of implementation frameworks and strategies to address implementation determinants, mechanisms, and outcomes [79, 80].

Regarding the psychometric properties of the individual items, most item difficulties were in the medium range. In the context of attitude measurements, a high item difficulty translates into low endorsement of the item. Medium difficulty is desirable as it is optimal to differentiate between respondents with different attitudes. Items of the subscales Fit, Feedback and Appeal received high approval (subscale means > 3.2), whereas items of the subscales Burden, Job Security and Divergence were less strongly endorsed (subscale means < 1.3). Removing any item would not have improved the internal consistency of the total scale and could result in poor content validity. While correlations between items and their subscales were at least in the medium range, item-whole correlations demonstrated considerable variability. Consistent with this, the subscales Fit, Monitoring, Burden and Feedback showed only moderate correlations with the EBPAS-36D total scale and only few subscales showed high inter-correlations, namely the subscales Appeal and Fit, Divergence and Limitations, Openness and Divergence, Appeal and Openness, and Job Security and Organizational Support. This accords well with previous results in US and Norwegian examinations of the EBPAS-36 that demonstrated high inter-correlations only between the Appeal and Organizational Support subscales (US) and between the Limitations and Divergence, and the Job Security and Organizational Support subscales (Norway) [51].

The internal consistency of the EBPAS-36D total scale obtained is good and comparable to those found for the US and Norwegian versions. The internal consistencies of the subscales ranged from acceptable to good, with the subscales Appeal, Divergence, Fit and Balance demonstrating the lowest internal consistencies, as seen in the Norwegian sample [51]. Due to their limited reliability, these subscales should be interpreted with caution. In consideration of the extreme brevity of the subscales (3 items), the overall reliability of the EBPAS-36D subscales can be considered high.

The CFA confirmed the 12-factor structure of the original EBPAS-36 by demonstrating adequate model fit. Nonetheless, two second-order factor structures, one derived by EFA in the present sample and one that was proposed by Rye et al. [41], showed even better model fits. Therefore, second-order models might map the actual underlying construct of attitudes toward EBP even better than the original factor structure. The four second-order constructs we found are: Positive alignment with EBP, consisting of the Openness, Appeal, Fit and Feedback subscales, Reservations toward EBP, consisting of the Divergence, Limitations and Balance subscales, Institutional Endorsement, consisting of the Job Security and Organizational Support subscales and Constraints by the institution, consisting of the Requirements, Monitoring and Burden subscales.

As expected, the EBPAS-36D showed high correlations with two other scales assessing attitudes toward the use of EBP, the direct and indirect measurement scales of attitudes of the ISP. This confirms the convergent validity of the scale. Accordingly, a high correlation was found between the EBPAS-36D and a global assessment of participant’s interest in EBP, a nine-item subjective self-rating of one’s interest in research on psychotherapy and clinical psychology, clinical guidelines, EBP and treatment manuals with high face validity. Moreover, the EBPAS-36D allowed incremental prediction of the intention to use EBP as assessed with the Behavioral Intention Scale of the ISP beyond gender, age and working in science.

Responders of the 50-item version of the scale commented on being annoyed and fatigued by answering the items [51]. This might result in response biases and missing answers, limiting the validity of the scale. The acceptability of the shorter EBPAS-36D appears to be high as indicated by a low amount of missing answers in the current study. Further, the EBPAS-36D is 28% shorter than the prior 50-item version and is consistent with calls for brief and pragmatic measures in implementation research [46]. With its 36 items and 12 first order subscales, the EBPAS-36D is a complex instrument and the reader may wonder about its feasibility. However, evidence-based practice itself is a complex construct and the attitudes of health professionals may vary on a number of dimensions. In our view, it is helpful to represent these dimensions in a detailed fashion on a measurement instrument. The most likely contexts of measurement will be evaluations of existing services and its stakeholders prior to the implementation of changes. In these contexts, a fine-grained assessment of the attitudes and views of the professionals may offer information about problem areas that might need attention in transformation processes (i.e. providing training, identifying obstacles). Given that the target group who will complete the questionnaire are health-professionals who are generally acquainted with such instruments, we feel confident that the length and complexity should not present any barriers.

In the present sample of German psychotherapists, higher age was associated with less favorable attitudes toward EBP. Consistent with this, licensed psychotherapists reported more negative attitudes compared to psychotherapists in training. While this result is in contrast to two previous studies reporting higher scores on the Requirements and Openness subscales of the EBPAS with increasing age [34, 35], it is in accordance with other studies [38–41]. As assumed by van Sonsbeek et al. [37], professionals may rate their own clinical experience higher than EBP with increasing age and experience. However, it should be noted that as yet, age effects may be confounded by cohort effects, since psychotherapy training underwent considerable changes in Germany over the last 30 years. Concerning sex differences, women reported more positive attitudes toward EBP in the present study. Sex differences were apparent for the total scale and the subscales Requirements, Appeal, Fit and Organizational Support. This result adds to other research demonstrating more positive attitudes toward EBP in women [34, 36–38, 41]. Still, these sex differences were not found consistently [26, 39, 40], which is why possible moderators should be investigated in future research.

As demonstrated in previous studies [27, 35], provider’s attitudes toward EBP were associated with organizational climate for the implementation of EBP. Since psychotherapists working in private practices were unable to rate the implementation climate of any organization or work group, those participants were asked to rate a parallel version of the ICS, capturing the implementation climate in the German health system. Interestingly, individuals’ attitudes toward EBP were not associated with their evaluation of the implementation climate of the health system. One reason for this might be that the German health system does not provide support for EBP to a similar extent as some organizations do, thus not leading to more positive attitudes toward EBP [28]. Another reason might be that psychotherapists with a positive attitude toward EBP are able to choose organizations with a better implementation climate for EBP or affect the implementation climate of the organization they work at, while they are probably unable to choose a health system according to its implementation climate or affect the implementation climate of the health system.

When interpreting the results of the present study, some limitations must be borne in mind. All data are based on self-reports in a cross-sectional online survey. A large proportion of the sample reported ever having worked in science and having a cognitive behavioral therapy approach. The findings refer to a convenience sample that is most likely self-selected for interest in EBP and not representative [81] of the population of mental health providers. This does not affect the evaluation of the psychometric properties of the EPBAS-36D; still future studies with representative samples should seek to confirm the results. Although the dropout rate in the present study can be considered as average for online surveys [82], a selection bias cannot be precluded with only particularly interested participants completing the survey [83]. The total number of items of the survey would have been significantly reduced if we have had the EBPI available when we planned our study. It would have been fruitful to use it instead of the ISP to investigate the convergent validity of the EBPAS-36D not only due to its smaller item number but because of the availability of a German version instrument that has been psychometrically examined [76]. In future studies, this well-suited questionnaire might be used to further validate the EBPAS-36D. Finally, although a definition of EBP preceded the questionnaire, some comments indicated that individual participants may have been uncertain about the exact meaning of EBP. Future research should assess the comprehensibility of the definition to ensure that all participants rate the same construct.

## Conclusions

The consideration of providers’ attitudes toward EBP in implementation research might inform about successful strategies to address their readiness to implement EBP, while in clinical practice this could point to important targets for addressing in training and supervision. Therefore, reliable instruments assessing attitudes toward EBP could be useful for researchers, training directors, and supervisors. Although further validating research is required, the present study confirms good psychometric properties and validity of a German version of the EBPAS-36 in a sample of psychotherapists. The proposed second-order model of attitudes toward EBP may initiate further research on the construct of attitudes toward EBP.

## Supplementary Information

**Additional file 1:** EBPAS-36D (German).

**Additional file 2:** EBPAS-36D Scoring Instructions (German).

**Additional file 3:** CHERRIES Checklist.

**Additional file 4:** Global assessments.

**Additional file 5:** Split sample *t*-tests.

**Additional file 6:** Rotated factor matrix.

## Data Availability

The datasets analyzed in the current study are available from the corresponding author on reasonable request.

## Abbreviations

AIC: Akaike information criterion
CBT: Cognitive behavioral therapy
CFA: Confirmatory factor analysis
CFI: Comparative Fit Index
CFIR: Consolidated Framework for Implementation Research
CHERRIES: Checklist for Reporting Results of Internet E-Surveys
COSMIN: COnsensus-based Standards for the selection of health Measurement INstruments
EBP: Evidence-based practices
EBPAS: Evidence-based Practice Attitudes Scale
EBPI: Evidence-based practice inventory
EFA: Exploratory factor analysis
EPIS: Exploration, preparation, implementation, sustainment framework
ICS: Implementation Climate Scale
ISP: Intention Scale for Providers
PCFI: Parsimony-adjusted Comparative Fit Index
RMSEA: Root Mean Square Error of Approximation
SRMR: Standardized Root Mean Squared Residual
STROBE: STrengthening the Reporting of OBservational studies in Epidemiology
US: United States
