# Supplementary material for: Cross-cultural adaption and psychometric investigation of the German version of the Evidence Based Practice Attitude Scale (EBPAS-36D)
Source: Health Res Policy Syst. 2021 Jun 2;19:90. doi: 10.1186/s12961-021-00736-8 (PMC8173815; doi:10.1186/s12961-021-00736-8)
Supplement: Supplementary file 1 — Additional file 1: EBPAS-36D (German). [file 12961_2021_736_MOESM1_ESM.docx]

**Einstellung zu evidenzbasierter Praxis Skala (EBPAS-36D)**

Die EBPAS-36D erfasst die Einstellungen von Gesundheitsdienstleistern, die im Bereich psychischer Störungen arbeiten, hinsichtlich der Implementierung von Innovationen und evidenzbasierten Verfahren (EBV) in Kontexten der psychologischen Versorgung und der Sozialdienste.

Die Items sind auf einer 5-stufigen Likert Skala von 0 „Überhaupt nicht“ bis 4 „In sehr großem Ausmaß“ einzuschätzen.

**Referenz**

Szota, K., Thielemann, J., Christiansen, H., Rye, M., Aarons, G. & Barke, A. (2021). Cross-cultural adaption and psychometric investigation of the German version of the Evidence Based Practice Attitude Scale (EBPAS-36D). *Health Research Policy and Systems.*

Bei Fragen kontaktieren Sie bitte:

Katharina Szota, szota@staff.uni-marburg.de

**Einstellung zu evidenzbasierter Praxis Skala (EBPAS-36D)**

Der folgende Fragebogen enthält Fragen zu Ihren Gefühlen in Bezug auf den Einsatz neuer Therapieformen, Interventionen oder Behandlungen.

Unter manualisierter Therapie versteht man dabei jede Intervention, die spezifische Richtlinien oder Komponenten hat, die in einem Handbuch beschrieben sind oder die strukturiert bzw. vorher festgelegt wurden und zu befolgen sind.

Bitte geben Sie an, in welchem Ausmaß Sie jedem Item zustimmen, indem Sie das untenstehenden Antwortformat nutzen.

| **0** | **1** | **2** | **3** | **4** |
| --- | --- | --- | --- | --- |
| **Überhaupt nicht** | **In einem geringen Ausmaß** | **In einem mittleren Ausmaß** | **In einem großen Ausmaß** | **In einem sehr großen Ausmaß** |

1. Ich nutze gern neue Therapieformen / Interventionen, um meinen Klient*innen zu helfen....0 1 2 3 4

2. Ich bin bereit, neue Therapieformen / Interventionen auszuprobieren, auch wenn ich dabei einem Behandlungsmanual folgen muss.................................................................................................0 1 2 3 4

3. Ich bin bereit, neue und verschiedene Therapieformen / Interventionen zu nutzen, die von Forscher*innen entwickelt wurden...............................................................................................0 1 2 3 4

4. Forschungsbasierte Therapieformen / Interventionen sind nicht klinisch nützlich..................0 1 2 3 4

5. Klinische Erfahrung ist wichtiger als die Nutzung manualisierter Therapien / Behandlungen...............................................................................................................................0 1 2 3 4

6. Ich würde keine manualisierte Therapie / Intervention benutzen.............................................0 1 2 3 4

*Zu den Fragen 7-15: Stellen Sie sich vor, dass Sie Training in einer Therapie oder Intervention erhalten, die Ihnen neu ist. Wie wahrscheinlich ist es, dass Sie sie umsetzen, wenn:*

7. diese in Ihren Augen „Sinn ergibt“?.........................................................................................0 1 2 3 4

8. diese von Ihrem*r Vorgesetzten gefordert wird?.....................................................................0 1 2 3 4

9. diese von Ihrem*r Einrichtung/Träger gefordert wird?............................................................0 1 2 3 4

10. diese von Ihrem Gesundheitssystem gefordert wird?.............................................................0 1 2 3 4

11. diese von Kolleg*innen genutzt wird, die damit zufrieden sind?...........................................0 1 2 3 4

12. Sie das Gefühl haben, genug Training erhalten zu haben, um sie korrekt einzusetzen?........0 1 2 3 4

13. Sie wüssten, dass es das Richtige für Ihre Klient*innen ist?..................................................0 1 2 3 4

14. Sie Mitspracherecht dabei hätten, wie Sie das evidenzbasierte Verfahren nutzen?...............0 1 2 3 4

15. diese zu Ihrem klinischen Ansatz passt?.................................................................................0 1 2 3 4

*Zu den Fragen 16-36: Bitte geben Sie das Ausmaß Ihrer Zustimmung mit den folgenden Aussagen an:*

16. Evidenzbasiertes Vorgehen ist nicht nützlich für Patient*innen mit multiplen Problemen...0 1 2 3 4

17. Evidenzbasiertes Vorgehen erlaubt keine individualisierte Behandlung...............................0 1 2 3 4

| **0** | **1** | **2** | **3** | **4** |
| --- | --- | --- | --- | --- |
| **Überhaupt nicht** | **In einem geringen Ausmaß** | **In einem mittleren Ausmaß** | **In einem großen Ausmaß** | **In einem sehr großen Ausmaß** |

18. Evidenzbasiertes Vorgehen hat einen zu engen Fokus...........................................................0 1 2 3 4

19. Ich arbeite lieber allein und ohne Aufsicht.............................................................................0 1 2 3 4

20. Ich möchte nicht, dass mir jemand über die Schulter schaut, während ich Gesundheitsleistungen erbringe.........................................................................................................................................0 1 2 3 4

21. Meine Arbeit muss nicht überwacht werden..........................................................................0 1 2 3 4

22. Ein positives Therapierergebnis ist eher eine Kunst als eine Wissenschaft...........................0 1 2 3 4

23. Therapie ist beides: eine Kunst und eine Wissenschaft..........................................................0 1 2 3 4

24. Meine allgemeine Kompetenz als Therapeut*in ist wichtiger als ein bestimmter Ansatz.....0 1 2 3 4

25. Ich habe keine Zeit, irgendetwas Neues zu lernen..................................................................0 1 2 3 4

26. Ich kann meine anderen Verpflichtungen nicht erfüllen........................................................0 1 2 3 4

27. Ich weiß nicht, wie ich evidenzbasiertes Vorgehen mit meinen Verwaltungsaufgaben vereinbaren soll.................................................................................................................................................0 1 2 3 4

28. Ein evidenzbasiertes Vorgehen zu erlernen wird mir helfen, meine Stelle zu behalten.........0 1 2 3 4

29. Ein evidenzbasiertes Vorgehen zu erlernen wird mir helfen, eine neue Stelle zu bekommen.....................................................................................................................................0 1 2 3 4

30. Ein evidenzbasiertes Vorgehen zu erlernen wird es leichter für mich machen, Arbeit zu finden............................................................................................................................................0 1 2 3 4

31. Ich würde ein evidenzbasiertes Vorgehen erlernen, wenn ich Fortbildungspunkte dafür erhielte..........................................................................................................................................0 1 2 3 4

32. Ich würde ein evidenzbasiertes Vorgehen erlernen, wenn es dafür Training gäbe................0 1 2 3 4

33. Ich würde ein evidenzbasiertes Vorgehen erlernen, wenn kontinuierliche Unterstützung zur Verfügung stünde..........................................................................................................................0 1 2 3 4

34. Ich bekomme gern Feedback zu meiner Arbeitsleistung........................................................0 1 2 3 4

35. Feedback zu erhalten, hilft mir, ein*e bessere*r Therapeut*in/Casemanager*in zu sein......0 1 2 3 4

36. Supervision zu erhalten, hilft mir, ein*e bessere*r Therapeut*in/Casemanager*in zu sein..0 1 2 3 4
