# Supplementary material for: Cross-cultural adaption and psychometric investigation of the German version of the Evidence Based Practice Attitude Scale (EBPAS-36D)
Source: Health Res Policy Syst. 2021 Jun 2;19:90. doi: 10.1186/s12961-021-00736-8 (PMC8173815; doi:10.1186/s12961-021-00736-8)
Supplement: Supplementary file 2 — Additional file 2: EBPAS-36D Scoring Instructions (German). [file 12961_2021_736_MOESM2_ESM.docx]

**Einstellung zu evidenzbasierter Praxis Skala (EBPAS-36D)**

**Auswertungshinweise**

Szota, K., Thielemann, J., Christiansen, H., Rye, M., Aarons, G. & Barke, A. (2021). Cross-cultural adaption and psychometric investigation of the German version of the Evidence Based Practice Attitude Scale (EBPAS-36D). *Health Research Policy and Systems.*

**Skala 1: Anforderungen**

Item 8 von Vorgesetzten gefordert

Item 9 von Einrichtung/Träger gefordert

Item 10 von Gesundheitssystem gefordert

**Skala 2: Attraktivität**

Item 7 ergibt Sinn

Item 11 Kolleg*innen damit zufrieden

Item 12 genug Training

**Skala 3: Offenheit**

Item 1 nutze gerne neue Interventionen

Item 2 bereit, Behandlungsmanual zu folgen

Item 3 von Forscher*innen entwickelt

**Skala 4: Abweichungen**

Item 4 nicht klinisch nützlich

Item 5 Klinische Erfahrung wichtiger

Item 6 Manualisierte Therapie

**Skala 5: Limitationen**

Item 16 Multiple Probleme

Item 17 Individualisiert

Item 18 Enger Fokus

**Skala 6: Passung**

Item 13 das Richtige für Ihre Klient*innen

Item 14 Mitspracherecht

Item 15 Ihr klinischer Ansatz

**Skala 7: Monitoring**

Item 19 Ohne Aufsicht

Item 20 über Schulter schaut

Item 21 muss nicht überwacht werden

**Skala 8: Ausgewogenheit**

Item 22 Eher Kunst als Wissenschaft

Item 23 Kunst und Wissenschaft

Item 24 allgemeine Kompetenz wichtiger

**Skala 9: Belastung**

Item 25 Keine Zeit

Item 26 Andere Verpflichtungen

Item 27 Verwaltungsaufgaben

**Skala 10: Arbeitsplatzsicherheit**

Item 28 hilft, Stelle zu behalten

Item 29 hilft, neue Stelle zu bekommen

Item 30 leichter, Arbeit zu finden

**Skala 11: Organisatorische Unterstützung**

Item 31 Fortbildungspunkte

Item 32 Training

Item 33 Kontinuierliche Unterstützung

**Skala 12: Feedback**

Item 34 bekomme gern Feedback

Item 35 Feedback hilft mir

Item 36 Supervision hilft mir

**Auswertung der Subskalen:**

Die Auswertung für jede Subskala erfolgt durch die Berechnung des Mittelwerts der Items, die (wie oben zugeordnet) auf einer Subskala laden. Wenn in Ihrem Datensatz Daten fehlen, können Subskalen berechnet werden, sofern nur ein Item pro Skala fehlt.

**Auswertung der Gesamtskala:**

Zur Auswertung der Gesamtskala (nicht zur Auswertung der Subskalenwerte) müssen die Items der Subskala 4 (Abweichungen), Subskala 5 (Limitationen), Subskala 7 (Monitoring), Subskala 8 (Ausgewogenheit), und Subskala 9 (Belastung) umgekehrt kodiert (0 => 4, 1 => 3, 3 => 1, 4 => 0) und die Subskalenwerte neu berechnet werden. Danach kann der Mittelwert der Subskalenwerte berechnet werden, um den EBPAS-36D Gesamtscore zu erhalten.

Bei Fragen kontaktieren Sie bitte Katharina Szota: szota@staff.uni-marburg.de
