# Supplementary material for: Cross-cultural adaption and psychometric investigation of the German version of the Evidence Based Practice Attitude Scale (EBPAS-36D)
Source: Health Res Policy Syst. 2021 Jun 2;19:90. doi: 10.1186/s12961-021-00736-8 (PMC8173815; doi:10.1186/s12961-021-00736-8)
Supplement: Supplementary file 4 — Additional file 4: Global assessments. [file 12961_2021_736_MOESM4_ESM.docx]

**Supplemental material 4**

**Table 1. Global assessments**

| **Interesse an EBP** | | **Interest in EBP** |
| --- | --- | --- |
|  | Bitte schätzen Sie mithilfe der Schieberegler ein, wie groß Ihr Interesse ist. *(Sehr gering – Sehr groß)* | Please use the sliders to estimate how great your interest is. *(Very low – Very high)* |
| 1 | Wie groß ist Ihr Interesse an der Forschung in Klinischer Psychologie? | How great is your interest in research in clinical psychology? |
| 2 | Wie groß ist Ihr Interesse an Studienergebnissen zur Effektivität von Behandlungsmethoden? | How great is your interest in study results on the effectiveness of treatment methods? |
| 3 | Wie groß ist Ihr Interesse an Studienergebnissen zur Effizienz von Behandlungsmethoden? | How great is your interest in study results on the efficiency of treatment methods? |
| 4 | Wie groß ist Ihr Interesse an evidenzbasierten Behandlungsmethoden? | How great is your interest in evidence-based treatment methods? |
|  | Bitte schätzen Sie mithilfe der Schieberegler ein, wie häufig Sie die folgenden Verhaltensweisen zeigen. *(Sehr selten – Sehr häufig)* | Please use the sliders to estimate how often you show the following behavior. *(Very rarely – Very often)* |
| 5 | Informieren Sie sich darüber, welche Behandlungsmethoden evidenzbasiert sind? | Do you inform yourself about which treatment methods are evidence-based? |
| 6 | Lesen Sie klinische Leitlinien (z.B. AWMF, DGPs o.a.) zur Behandlung von psychischen Störungen? | Do you read clinical guidelines (e.g. AWMF, DGPs, etc.) for the treatment of mental disorders? |
| 7 | Lesen Sie wissenschaftliche Publikationen? | Do you read scientific publications? |
| 8 | Setzen Sie evidenzbasierte Behandlungsmethoden ein? | Do you use evidence-based treatment methods? |
| 9 | Kaufen Sie aktuelle Behandlungsmanuale? | Do you buy current treatment manuals? |
| **Ehrlichkeit der Angaben in der Umfrage** | | **Honesty of survey responses** |
|  | Wie ehrlich haben Sie die Fragen dieser Studie beantwortet? *(Nicht besonders ehrlich – Sehr ehrlich)* | How honestly did you answer the questions of this study answered? *(Not particularly honest – Very honest)* |
| **Tendenz zur sozialen Erwünschtheit** | | **Tendency towards social desirability** |
|  | Hat soziale Erwünschtheit bei der Befragung eine Rolle gespielt? *(In sehr geringem Ausmaß – In sehr großem Ausmaß)* | Did social desirability play a role in the survey? *(To a very small extent – To a very large extent)* |
