# Supplementary material for: Cross-cultural adaption and psychometric investigation of the German version of the Evidence Based Practice Attitude Scale (EBPAS-36D)
Source: Health Res Policy Syst. 2021 Jun 2;19:90. doi: 10.1186/s12961-021-00736-8 (PMC8173815; doi:10.1186/s12961-021-00736-8)
Supplement: Supplementary file 5 — Additional file 5: Split sample t-tests. [file 12961_2021_736_MOESM5_ESM.docx]

**Supplemental material 5**

**Table 1. Independent t-tests for split samples on age, gender, EBPAS-36D Total Score and Subscales**

|  | EFA Sample (*n* = 296) | | CFA Sample (*n* = 278) | | Levene test | | Independent t-tests | | |
| --- | --- | --- | --- | --- | --- | --- | --- | --- | --- |
|  | Mean | SD | Mean | SD | *F* | *p* | *T* | df | *p* |
| Age | 35.75 | 10.26 | 36.75 | 11.66 | 3.58 | .059 | -1.09 | 572 | .274 |
| Gender | 1.14 | 0.35 | 1.18 | 0.40 | 8.61 | .003 | -1.44 | 550.29 | .150 |
| EBPAS-36D Total Score | 2.58 | 0.46 | 2.57 | 0.41 | 0.93 | .335 | 0.32 | 571 | .748 |
| EBPAS-36D Requirements | 2.20 | 0.97 | 2.20 | 0.93 | 0.73 | .392 | -0.04 | 572 | .969 |
| EBPAS-36D Appeal | 3.28 | 0.64 | 3.26 | 0.60 | 0.85 | .357 | 0.33 | 572 | .743 |
| EBPAS-36D Openness | 2.87 | 0.74 | 2.90 | 0.71 | 0.74 | .389 | -0.45 | 572 | .653 |
| EBPAS-36D Divergence | 1.23 | 0.76 | 1.30 | 0.78 | 0.04 | .852 | -1.01 | 572 | .311 |
| EBPAS-36D Limitations | 1.68 | 0.93 | 1.76 | 0.94 | 0.05 | .816 | -0.97 | 572 | .334 |
| EBPAS-36D Fit | 3.49 | 0.61 | 3.51 | 0.54 | 1.12 | .291 | -0.36 | 572 | .719 |
| EBPAS-36D Monitoring | 1.97 | 1.06 | 1.94 | 0.96 | 4.07 | .044 | 0.38 | 571.37 | .704 |
| EBPAS-36D Balance | 2.17 | 0.80 | 2.27 | 0.79 | 0.76 | .383 | -1.62 | 572 | .106 |
| EBPAS-36D Burden | 1.12 | 0.95 | 1.03 | 0.87 | 3.22 | .073 | 1.10 | 572 | .270 |
| EBPAS-36D Job security | 1.66 | 0.85 | 1.70 | 0.84 | 0.01 | .934 | -0.46 | 572 | .648 |
| EBPAS-36D Org. support | 2.47 | 1.06 | 2.33 | 1.11 | 0.48 | .487 | 1.58 | 572 | .114 |
| EBPAS-36D Feedback | 3.23 | 0.66 | 3.29 | 0.66 | 0.03 | .868 | -1.02 | 572 | .308 |

Notes. EBPAS-36D: German Translation of Evidence-Based Practice Attitudes Scale * *p* < .05 ** *p* < .001
