# Supplementary material for: Cross-cultural adaption and psychometric investigation of the German version of the Evidence Based Practice Attitude Scale (EBPAS-36D)
Source: Health Res Policy Syst. 2021 Jun 2;19:90. doi: 10.1186/s12961-021-00736-8 (PMC8173815; doi:10.1186/s12961-021-00736-8)
Supplement: Supplementary file 6 — Additional file 6: Rotated factor matrix. [file 12961_2021_736_MOESM6_ESM.docx]

**Supplemental material 6**

**Table 1. EFA: Rotated factor pattern matrix**

| Component/  Item (Original subscale) | 1  Positive alignment with EBP | 2  Reservations towards EBP | 3  Institutional endorsement | 4  Constraints by the institution | 5  Monitoring | 6  Burden |
| --- | --- | --- | --- | --- | --- | --- |
| 7 (Appeal) | **.844** |  |  |  |  |  |
| 13 (Fit) | **.818** |  |  |  |  |  |
| 15 (Fit) | **.754** |  |  |  |  |  |
| 14 (Fit) | **.659** |  |  |  |  |  |
| 12 (Appeal) | **.646** |  |  |  |  |  |
| 35 (Feedback) | **.602** |  |  |  |  |  |
| 11 (Appeal) | **.524** |  |  |  |  |  |
| 34 (Feedback) | **.488** |  |  |  |  |  |
| 36 (Feedback) | **.418** | .335 |  |  |  |  |
| 1 (Openness) | **.400** |  |  |  |  |  |
| 3 (Openness) | **.357** | -.335 |  |  |  |  |
| 18 (Limitations) |  | **.773** |  |  |  |  |
| 17 (Limitations) |  | **.715** |  |  |  |  |
| 5 (Divergence) |  | **.683** |  |  |  |  |
| 16 (Limitations) |  | **.611** |  |  |  |  |
| 2 (Openness) |  | **-.559** |  |  |  |  |
| 6 (Divergence) | -.361 | **.556** |  |  |  |  |
| 22 (Balance) |  | **.535** |  |  |  |  |
| 24 (Balance) |  | **.503** |  |  |  |  |
| 4 (Divergence) |  | **.415** |  |  |  |  |
| 23 (Balance) |  | **.410** |  |  |  |  |
| 29 (Job Security) |  |  | **.969** |  |  |  |
| 30 (Job Security) |  |  | **.956** |  |  |  |
| 28 (Job Security) |  |  | **.754** |  |  |  |
| 32 (Org. Support) |  |  | **.416** |  |  |  |
| 33 (Org. Support) |  |  | **.349** |  |  |  |
| 31 (Org. Support) |  |  | **.343** |  |  |  |
| 9 (Requirements) |  |  |  | **.946** |  |  |
| 8 (Requirements) |  |  |  | **.882** |  |  |
| 10 (Requirements) |  |  |  | **.735** |  |  |
| 19 (Monitoring) |  |  |  |  | **.815** |  |
| 20 (Monitoring) |  |  |  |  | **.695** |  |
| 21 (Monitoring) |  |  |  |  | **.549** |  |
| 26 (Burden) |  |  |  |  |  | **.841** |
| 27 (Burden) |  |  |  |  |  | **.715** |
| 25 (Burden) |  |  |  |  |  | **.693** |

Notes. *n* = 296.
